# Bidirectional Relationship between Adolescent Gender Egalitarianism and Prosocial Behavior

**DOI:** 10.3390/bs14010033

**Published:** 2024-01-02

**Authors:** Xinyuan Fu, Ruoran Fu, Yanping Chang, Zhixu Yang

**Affiliations:** 1 Department of Psychology, School of Sociology and Psychology, Central University of Finance and Economics, Beijing 100081, China; 2 School of Labor Economics, Capital University of Economics and Business, Beijing 100070, China

**Keywords:** gender egalitarianism, prosocial behavior, multi-group cross-lagged panel model, adolescence

## Abstract

This study investigates the bidirectional associations between gender egalitarianism and prosocial behavior in adolescents, and the moderating effect of gender in the associations, as well as gender differences and longitudinal changes in both. We recruited 543 Chinese adolescents (284 girls, 259 boys; mean age at Time 1 = 11.27 years) and collected three waves of data measuring gender egalitarianism and prosocial behavior at one-year intervals. According to the results, girls expressed greater gender egalitarianism than boys did; girls reported more prosocial behavior than boys in the sixth grade, but there were no significant gender differences in the seventh and eighth grades. Adolescents’ gender egalitarianism stayed stable from the sixth to the seventh grade then increased from the seventh to the eighth grade, and there was a decrease in prosocial behavior from the sixth to the seventh grade. More importantly, the results of the multi-group cross-lagged panel model revealed that adolescents’ gender egalitarianism in the previous year positively predicted prosocial behavior in the next year, and vice versa; such bidirectional associations equally applied to boys and girls. These findings add to the knowledge of adolescent gender egalitarianism and prosocial behavior, and the dynamic interplay between the two.

## 1. Introduction

Adolescence is a key period when personal values and prosocial behaviors develop and diversify [1,2]. Meanwhile, the development of prosocial behavior in adolescence is a critical inflection period for social adjustment [3]. Thus, investigating the prosocial development of adolescents is essential for individual developmental trajectories. Understanding the impact of values on prosocial behavior facilitates making more effective policies to improve adolescents’ prosocial behavior [4]. In fact, prosocial behavior is demonstrated to be shaped by personal values in adolescence, such as prosocial values, materialistic values, and cultural values, while previous studies have also found that adolescent prosocial behavior can reinforce the above values [5,6,7]. However, other crucial values, such as gender egalitarianism, have been neglected in value–prosocial development bidirectional linkages. Recent studies indicate that adolescents show increasing differentiation between outgroups and ingroups [3], of which gender is an obvious basis of group differentiation [8,9], which may become a latent premise of gender bias [10], and thus gender inequality. It is satisfying to note that prosocial behavior is an important way to eliminate bias toward outgroups [11]. Under this context, to establish social connections between different genders and create a more gender-equal society, it is necessary to understand the developmental trajectories of gender egalitarianism and prosocial behavior.

Gender egalitarianism is a value system that supports men and women having equal roles, rights, and responsibilities [12]. Interest in gender egalitarianism remains high among political psychologists, because public support for gender egalitarianism could reduce societal-level structural gender inequality [13,14] and predict individual-level women-favoring political attitudes and engagement [15,16,17]. However, a significant omission in the literature could be adolescent gender egalitarianism from a developmental perspective. Limited studies that have focused on adolescent samples point to the role of gender egalitarianism on adolescent political attitudes [18] and intergenerational continuity as a precursor of adolescent gender egalitarianism [19]. Nonetheless, the longitudinal associations between adolescent gender egalitarianism and prosocial behavior are unclear. In addition, psychology has a prolonged tradition of investigating the impact of an agent’s gender on both gender egalitarianism and prosocial behavior [20,21], and understanding whether gender disparity among the existing associations could provide policy makers with guidance for making gender-sensitive policy [22]. This tradition and practical significance necessitate exploring gender’s potential moderating role in the longitudinal associations between adolescent gender egalitarianism and prosocial behavior.

To explore more angles for promoting the positive development of adolescent gender egalitarianism and prosocial behavior, as well as the virtuous circle between these two variables, an investigation of the longitudinal interaction between gender egalitarianism and prosocial behavior is necessary. Therefore, the present study’s goals are threefold. First, it investigates the gender differences and longitudinal changes in adolescent gender egalitarianism and prosocial behavior from a developmental perspective. Second, it examines the bidirectional relationship between adolescent gender egalitarianism and prosocial behavior. Third, it explores the moderating effect of adolescent gender in the longitudinal linkages between gender egalitarianism and prosocial behavior.

### 1.1. Gender Differences and Longitudinal Changes in Gender Egalitarianism and Prosocial Behavior

Previous studies have demonstrated a pancultural phenomenon of adult females reporting higher gender egalitarianism than males [18,23]. Hegemonic gender cultures make people believe that men and women have different interest structures [24]. Under such a cultural context, males are less inclined to hold the belief that they will benefit from gender equality than females [12,25]. Fortunately, males with degrees in education are more egalitarian than other men, and even than women with degrees in education [26], which indicates that gender equality could be achieved by education. Despite this, males usually have lower gender egalitarianism than females. Regarding longitudinal changes in gender egalitarianism, a longitudinal study found that gender egalitarianism increased as late adolescents entered emerging adulthood [27]. This change aligns with the increasing exposure to ideas and situations that encourage gender equality (e.g., increased education and labor market participation) in the transition from adolescence to adulthood [25]. However, recent research about gender differences in values across cultures indicates that universalism, which is broader and not limited to gender egalitarianism, shows no significant change in longitudinal patterns [28]. Given the little and mixed empirical evidence on the gender differences and longitudinal changes in adolescent gender egalitarianism, the current research attempts to investigate both in Chinese adolescents. 

Meanwhile, as for gender differences in prosocial behavior, most studies showed that boys exhibited lower levels of prosocial behavior than girls [29,30], whereas a meta-analysis showed that gender differences in various types of prosocial behavior were inconclusive; for instance, girls reported more compliant but fewer public prosocial behaviors than boys [31]. Regarding the adolescent prosocial development trajectory, previous research yielded inconsistent results, demonstrating that prosocial behavior remained stable from the fourth through twelfth grades [32], which even received support from neural evidence [33], or decreased from the seventh through eleventh grades with a slight rebound in the twelfth grade [34]. Given the contradictory evidence on gender differences and longitudinal changes in adolescent prosocial behavior, the present research tries to investigate both using a sample of Chinese adolescents.

### 1.2. Role of Gender Egalitarianism in Prosocial Behavior 

The hypothesis regarding the role of adolescent gender egalitarianism in prosocial behavior can be formulated using basic value theory and social dominance theory. On the one hand, gender egalitarianism can be deemed as a peculiar form of universalism values, which refer to “understanding, appreciation, tolerance, and protection regarding the welfare of all people and nature” [2]. Universalism has been proved to play a positive role in a variety of prosocial behaviors, such as philanthropic donation [35] and prosocial behavior based on standards of justice [36]. According to Schwartz’s [37] basic value theory, when people realize resources are scarce, universalism values such as gender egalitarianism are socialized and arise, promoting prosocial behaviors such as sharing resources. Furthermore, some peculiar universalisms, such as speciesism, which advocates equality among different species, could lead to more prosocial behavior [38]. Following the above logic, gender egalitarianism, as a peculiar universalism, is compatible with prosocial behavior such that acting prosocially can help to achieve the goals of gender egalitarianism [37]. Hence, adolescent gender egalitarianism and prosocial behavior are likely to have a positive relationship.

On the other hand, gender egalitarianism can be deemed as the opposite of gender-related social dominance orientation (SDO), which refers to the degree to which people support gender inequalities [39,40]. Individuals with high gender-related SDO tend to maintain their advantages, so they are more reluctant to allocate resources to others and exhibit less prosocial behaviors [41,42,43]. To some extent, prosocial behavior refers to the generous allocation of resources to people in need. Many studies even use resource allocation tasks to measure individuals’ prosocial motivation [44,45]. Hence, it is logical to assume that adolescents who endorse gender egalitarianism (i.e., argue against gender-related SDO) would be more likely to perform prosocial behavior than their counterparts who object to gender egalitarianism. 

### 1.3. Role of Prosocial Behavior in Gender Egalitarianism

Self-perception theory can provide a theoretical guidance for understanding the role of prosocial behavior in adolescents’ gender egalitarianism. This theory argues that people form attitudes and preferences via interpreting the meanings of their own behaviors [46]. Prosocial behavior has a positive impact on empathy, which would make individuals more easily perceive others’ feelings [47]. Thus, prosocial interactions with others can help adolescents become more aware that both men and women may have moments when they need help, and have more positive relationships with others, whether of the same or opposite gender [48]. In this manner, adolescents with greater levels of prosocial behavior could be more likely to view males and females as more similar than different, and consequently hold higher gender egalitarianism.

Apart from this, when people repeatedly engage in specific actions, it tends to shape their attitudes and beliefs about those actions. This fundamental logic has been applied widely to help citizens develop healthy and positive values, such as environmentally friendly values [49], and has also been examined in the field of developmental psychology. For instance, prosocial behavior toward powerful people was found to be positively associated with power distance value one year later among Chinese adolescents [5]. Helping strangers was positively correlated with adolescents’ subsequent benevolent values after one year [6]. When expressing more prosocial behaviors toward others regardless of gender, adolescents might view equality between genders as a natural part of the social order, and develop positive views about gender equality. Together with the above theoretical reasoning, it is rational to expect a positive association between adolescent prosocial behavior and subsequent gender egalitarianism. 

### 1.4. Adolescent Gender as a Moderator

To have a better understanding of the associations between adolescent gender egalitarianism and prosocial behavior, we sought to test adolescent gender as a moderator. From an evolutionary perspective, females place great weight on their prospective mates’ social and economic status [50]. This sexual selection process causes males to have a higher social dominance orientation and lower egalitarianism toward the outgroup than females [38,42,51]. At the same time, according to social role theory [52], social role norms require women to help others in almost all situations and be more empathetic and less competitive [53], while men are expected to strive for achievement and success in both competitive and cooperative tasks [54]. These social norms are introduced throughout the development of adolescents [55]. Empirically, a great number of studies have revealed gender differences regarding adolescent gender egalitarianism and prosocial behavior, as mentioned earlier [23,29]. However, it is unclear if adolescent gender moderates the associations between gender egalitarianism and prosocial behavior. Therefore, this study aims to test if the associations between adolescent gender egalitarianism and prosocial behavior differ across genders. 

### 1.5. The Present Study

This research investigates the gender differences and longitudinal changes in adolescent gender egalitarianism and prosocial behavior, and examines the bidirectional relationship between the two, along with the moderating effect of gender. We hypothesized that gender egalitarianism in the prior year would be positively associated with prosocial behavior in the following year (Hypothesis 1), and earlier prosocial behavior would be positively predictive of subsequent gender egalitarianism (Hypothesis 2). 

Given the mixed findings about gender differences and longitudinal trajectories of gender egalitarianism and prosocial behavior amid adolescence, and the exploratory nature of our studying the moderating effect of adolescent gender, we did not compose specific hypotheses for these two research questions. To respond to the research questions, a sample of Chinese adolescents was recruited and three waves of data at one-year intervals were collected; a multi-group cross-lagged panel model was performed to examine the above hypotheses with adolescent age and subjective household economic status as the control variables. Given the present study primarily investigates the prospective effects of between-person differences in gender egalitarianism and prosocial behavior, combined with the advantage of the cross-lagged panel model (CLPM) in testing prospective between-person effects, the CLPM was selected in data analysis. 

## 2. Method

### 2.1. Participants

A total of 543 first-year students from four middle schools in Fangshan District, Beijing, China were recruited at Time 1 in 2015. There were 284 girls and 259 boys and the mean age was 11.27 years old (*SD*_age_ = 0.51). In 2016, 449 of them participated in Time 2. The attrition rate was 17.31% as 94 participants failed to attend due to commitments. In 2017, 417 adolescents took part in Time 3. As 8 of the 417 participants missed the survey at Time 2, 409 students participated in all three waves of the survey. It should be noted that the first-year students were actually sixth-graders, since their educational system includes five years for elementary school and four years for middle school. 

In terms of the initial sample (*n* = 543), 22.10% of fathers and 25.60% of mothers had a middle school diploma or lower, 33.15% of fathers and 31.31% of mothers possessed a high school diploma or equivalent educational level, 16.21% of fathers and 20.07% of mothers had a college degree, 17.50% of fathers and 14.73% of mothers were bachelor holders, 3.31% of fathers and 2.21% of mothers were master or higher degree holders, and 7.73% of fathers’ and 6.08% of mothers’ data were missing. About 16.94% of adolescents had a monthly family income less than CNY 5000 (equivalent to about USD 774 in 2015), 57.46% reported between CNY 5001 and CNY 10,000 (equivalent to about USD 1549), 11.05% reported between CNY 10,001 and CNY 15,000 (equivalent to about USD 2323), 3.87% reported above CNY 15,000, and 10.68% of data were missing. In terms of subjective household economic status, 0.55% of participants thought their family was extremely poor, 4.97% thought their family was poor, 70.17% thought their family was average, 17.86% thought their family was rich, 0.37% thought their family was extremely rich, and 6.08% of data were missing.

### 2.2. Procedure 

The Institutional Review Board of Central University of Finance and Economics approved the procedure. Convenience sampling was used. We informed the headmasters of the target schools about the purpose of our study and obtained their permission before collecting data. The first-year students were informed that the survey was about their interactions with others and their responses would be kept confidential. The students who agreed to participate in the survey provided written informed consent from their parents. The survey took about 15 min at each time point, and it was carried out in quiet classrooms with a research assistant present to keep order and collect data in the classroom. Following the survey, participants received gifts like pens and were debriefed. Two English graduates were hired to translate and back-translate the originally English measures (i.e., gender egalitarianism and prosocial behavior) to ensure accuracy [56].

### 2.3. Measures

#### 2.3.1. Gender Egalitarianism

Gender Egalitarianism was assessed using a 5-item measure [57]. Items can be seen in Table 1. Responses ranged from 1 (strongly disagree) to 5 (strongly agree). Greater gender egalitarianism was indicated by higher mean values after one of the items was reverse-coded (McDonald’s omega = 0.71 at Time 1; 0.80 at Time 2; 0.81 at Time 3). To identify the measurement invariance across gender, we developed an unconstrained multi-group model of confirmatory factor analysis with three latent variables created (one per time point). Then, a constrained multi-group model with factor loadings set equal across gender was conducted. Further, a constrained multi-group model with both factor loadings and intercepts set equal across gender was conducted. Based on the guidelines given by Cheung and Rensvold [58] and Chen [59], if CFI changes by no more than 0.01 and RMSEA changes by no more than 0.015 for the constrained model relative to the unconstrained one, invariance is achieved. Therefore, strong invariance was not achieved. In this case, partial strong invariance was achieved after setting the intercepts of six items unconstrained (see Table 2). To identify the measurement invariance across time, the same analytical steps were implemented and partial strong invariance was achieved (only one item’s intercept at Time 3 was unconstrained). Factor loadings ranged from 0.33 to 0.97. 

#### 2.3.2. Prosocial Behavior 

Prosocial behavior was assessed using a modified version of the kindness and generosity subscale of the Values in Action Inventory of Strengths [60]. This measure included seven items. Items can be seen in Table 1. Each item was rated on a 5-point scale (1 = not like me at all, 5 = very much like me). Average values were calculated to indicate levels of prosocial behavior (McDonald’s omega = 0.90 at Time 1; 0.93 at Time 2; 0.94 at Time 3). The measurement invariances across gender and time were tested, and both achieved strong invariance (see Table 2). Factor loadings ranged from 0.47 to 0.91.

### 2.4. Data Analyses

First, missing data and attrition analyses were carried out. Second, descriptive statistics and correlation analyses on the study variables were performed using SPSS (version 26). Third, the repeated measures ANOVA was executed to explore the gender and longitudinal differences in adolescent gender egalitarianism and prosocial behavior. Fourth, Mplus (version 8.3) was used to perform confirmatory factor analysis and to test measurement invariance across gender and time for each latent variable. Fifth, multi-group cross-lagged panel models were employed to test the research hypotheses. It needs to be noted that full-information maximum likelihood (FIML) was used for missing data estimation and the maximum likelihood (ML) estimation was used for structural equation modeling. 

## 3. Results

### 3.1. Missing Data and Attrition Analyses

The missing values of the variables ranged from 0% to 23.76% for the study sample (*n* = 543), resulting in a total of 12.69% missing data. Little’s Missing Completely at Random (MCAR) Test suggested a significant deviation from MCAR, χ^2^ (2152) = 2533.51, *p* < 0.001. Next, to identify if the 409 retained participants were different from the 134 respondents who dropped out at Times 2 and/or 3, *t*-tests and a chi-square test were executed. No differences in gender and monthly family income were found between these two groups (*p* = 0.311; *p* = 0.244). However, the participants who quit at Times 2 and/or 3 were older (*p* = 0.023), had parents with lower educational levels (*p* < 0.001; *p* < 0.001), and reported lower subjective household economic status (*p* = 0.001). There were no significant differences between these two groups in terms of Time 1’s gender egalitarianism (*p* = 0.615) and prosocial behavior (*p* = 0.109). In sum, the sample did not experience attrition with respect to gender, monthly family income, gender egalitarianism, or prosocial behavior, though became younger and more biased toward better-educated families, and had higher subjective household economic status. 

### 3.2. Descriptive Statistics and Correlations

The descriptive statistics and correlations for the key variables are shown in Table 3. Gender egalitarianism assessed at Times 1, 2, and 3 correlated positively with each other, and so did prosocial behavior. Moreover, gender egalitarianism assessed at Times 1, 2, and 3 correlated positively with prosocial behavior assessed at Times 1, 2, and 3.

### 3.3. Gender Differences and Longitudinal Changes

Girls reported a higher level of gender egalitarianism than boys; the estimated marginal mean for girls was 4.03 (*SE* = 0.04), and that for boys was 3.75 (*SE* = 0.04), *F*(1, 403) = 20.73, *p* < 0.001, η_p_^2^ = 0.05. For longitudinal changes, the adolescents had a higher level of gender egalitarianism at Time 3 than that at Times 1 and 2, with no difference between Times 1 and 2, *F*(2, 806) = 10.40, *p* < 0.001, η_p_^2^ = 0.03 (see the means and standard deviations in Table 1). In other words, the adolescents’ gender egalitarianism stayed stable from the sixth to the seventh grade, and then increased from the seventh to the eighth grade. The interaction effect between gender and time point was not significant, *F*(2, 806) = 2.87, *p* = 0.057. 

With regard to prosocial behavior, the main effect of gender was not significant; the estimated marginal mean for girls was 4.24 (*SE* = 0.04), and that for boys was 4.15 (*SE* = 0.04), *F*(1, 405) = 2.24, *p* = 0.136. As for the longitudinal changes, the adolescents had a lower level of prosocial behavior at Time 2 than that at Time 1, but the differences between Times 1 and 3 and between Times 2 and 3 were both not significant, *F*(2, 810) = 3.34, *p* = 0.036, η_p_^2^ = 0.01 (see the means and standard deviations in Table 1). In other words, there was a decrease in the adolescents’ prosocial behavior from the sixth to the seventh grade. Gender and time point had a significant interaction effect, *F*(2, 810) = 3.09, *p* = 0.046, η_p_^2^ = 0.01. The girls reported more prosocial behavior than boys (*M*_girls_ = 4.35, *SD*_girls_ = 0.66; *M*_boys_ = 4.15, *SD*_boys_ = 0.90) in the sixth grade, but there were no significant gender differences in the seventh (*M*_girls_ = 4.17, *SD*_girls_ = 0.75; *M*_boys_ = 4.11, *SD*_boys_ = 0.77) and eighth (*M*_girls_ = 4.20, *SD*_girls_ = 0.70; *M*_boys_ = 4.19, *SD*_boys_ = 0.83) grades. 

### 3.4. Multi-Group Cross-Lagged Panel Model

An unconstrained multi-group cross-lagged panel model was performed first. In this model, we included the three waves of adolescents’ gender egalitarianism and prosocial behavior to test the cross-lagged effects. Potential gender differences were tested via multi-group modeling and comparison. Age and subjective household economic status were included as the control variables. In addition, attrition or not was included in the model as a covariate. The unconstrained multi-group cross-lagged panel model fit the data adequately, χ^2^ (1424) = 2140.09, *p* < 0.001, CFI = 0.932, TLI = 0.929, RMSEA = 0.043. Then, a constrained multi-group cross-lagged panel model was performed, in which the autoregressive and cross-lagged paths were set equal across both gender and time. This constrained model fit the data adequately, χ^2^ (1436) = 2154.91, *p* < 0.001, CFI = 0.931, TLI = 0.930, RMSEA = 0.043. According to the guidelines proposed by Cheung and Rensvold [58] and Chen [59], the equivalence of the constrained and unconstrained models (ΔCFI = 0.001, ΔRMSEA = 0) indicated that fixing the multi-group, autoregressive, and cross-lagged paths was defensible.

**Figure 1:**
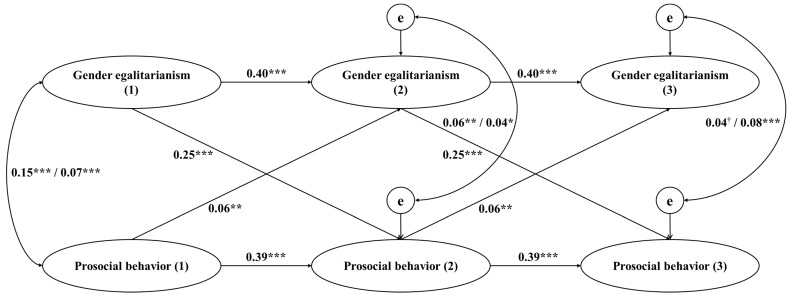
Cross-lagged effects of boys’ and girls’ gender egalitarianism and prosocial behavior. *Note.* Values presented are unstandardized coefficients. Values with a slash represent paths that vary freely across gender (values before the slash are for boys); otherwise the values are identical across gender. Control variables are omitted for parsimony. χ^2^ (1436) = 2154.91, *p* < 0.001, CFI = 0.931, TLI = 0.930, RMSEA = 0.043. (1) = Time 1; (2) = Time 2; (3) = Time 3. ^†^ *p* = 0.058, * *p* < 0.05, ** *p* < 0.01, *** *p* < 0.001.

As shown in Figure 1, for both boys and girls, gender egalitarianism at Time 1 was significantly correlated with prosocial behavior at Time 2 (*B* = 0.25, *p* < 0.001) and gender egalitarianism at Time 2 was significantly associated with prosocial behavior at Time 3 (*B* = 0.25, *p* < 0.001). Moreover, both boys’ and girls’ prosocial behavior at Time 1 was positively predictive of gender egalitarianism at Time 2 (*B* = 0.06, *p* = 0.005) and their prosocial behavior at Time 2 was positively predictive of gender egalitarianism at Time 3 (*B* = 0.06, *p* = 0.005). The control variables had no significant effects.

## 4. Discussion

This study investigated the gender differences and longitudinal changes in Chinese adolescent gender egalitarianism and prosocial behavior, and firstly examined the bidirectional relationship between the two, along with the moderating effect of gender. 

With regard to the gender differences and longitudinal changes in gender egalitarianism, we found girls held greater gender egalitarianism than boys, which is in line with previous studies on gender differences regarding gender egalitarianism across various cultures [18,20,23]. Meanwhile, adolescents’ gender egalitarianism stayed stable from the sixth to the seventh grade and then increased from the seventh to the eighth grade, which supports previous observations in Germany [61]. Concerning prosocial behavior, previous findings about gender differences on the development of prosocial behavior in adolescents are mixed. For example, Xiao et al. [31] showed that there were smaller gender differences in prosocial behaviors through adolescence, whereas some studies have indicated that the gender gap became larger or existent at least during adolescence [30,34]. The present study supports Xiao et al. [31], showing that girls reported more prosocial behavior than boys in the sixth grade, but there were no significant gender differences in the seventh and eighth grades. 

Regarding the longitudinal interplay of gender egalitarianism and prosocial behavior, previous studies have investigated either the longitudinal development of gender egalitarianism [61] or prosocial behavior [3,5,6,7]. To our knowledge, this is the first study to uncover the longitudinal relationship between them. Owing to the intricate link between value and behavior, investigating the associations between gender egalitarianism and prosocial behavior is beneficial for exploring more angles to promote their development, as well as this virtuous cycle. In brief, the results indicate that prosocial behavior positively predicted the presence of gender egalitarianism over time, and vice versa, while there was no gender difference in this bidirectional relationship.

### 4.1. Gender and Longitudinal Differences in Gender Egalitarianism and Prosocial Behavior

The results showed that girls held higher levels of gender egalitarianism than boys, which accords with previous studies [18,20,23]. According to social dominance theory, the dominant side would possess more anti-egalitarianism to sustain their dominance and the non-dominant side would possess more egalitarianism [50]. Given that females are still in a disadvantaged position across different situations, such as leadership [62] and selection of deanship in colleges [63], it is reasonable that females have higher levels of gender egalitarianism than males. Notably, the findings above were found in adults. So far, only a recent study has found that adolescents showed gender differences in gender egalitarianism in Germany [61], and the present study showed a consistent finding among Chinese adolescents. Moreover, we found that adolescents’ gender egalitarianism stayed stable from the sixth to the seventh grade and then increased from the seventh to the eighth grade. This trend is aligned with increasing exposure to ideas and situations that encourage gender equality (e.g., increased education) as adolescents age [25,61], as well as the development of cognitive ability facilitating them to consider their sex roles more flexibly and eliminate sex biases gradually [64].

Concerning prosocial behavior, we found that girls reported more prosocial behavior than boys in the sixth grade, but there were no significant gender differences in the seventh and eighth grades. In other words, the gender disparity in prosocial behavior gradually disappeared among Chinese adolescents from early to middle adolescence, which is inconsistent with some previous studies [30,34]. This inconsistency could be explained jointly with another interesting finding in the present study; that is, there was a decrease in prosocial behavior from the sixth to the seventh grade and no other longitudinal differences were found. For Chinese adolescents, the transition from the sixth to the seventh grade is a critical period in which they experience increased academic burdens, life challenges, and mental health issues, regardless of gender; such transition causes them to focus more on their own needs and reduces their time available to help others. Hence, both boys and girls in the present study kept relatively low and stable levels of prosocial behavior in the seventh to the eighth grade. 

### 4.2. Interrelationship between Adolescent Gender Egalitarianism and Prosocial Behavior 

According to the results, adolescents’ gender egalitarianism was positively predictive of subsequent prosocial behavior. This finding supports both basic value theory and social dominance theory. As a peculiar form of universalism values and the opposite of gender-related social dominance orientation, gender egalitarianism emphasizes the equal rights, roles, and responsibilities of different genders [12]. Thus, gender egalitarianism naturally motivates individuals to be concerned about the equal distribution of resources across genders and conduct prosocial behaviors toward either males or females at a disadvantage, which is similar with speciesism, a belief about equal rights across different species [38]. In the study sample, higher gender egalitarianism of adolescents was associated with more prosocial behaviors, highlighting the value-to-behavior effect during adolescence. Moreover, the compatibility in nature between gender egalitarianism and prosocial behavior, such as caring and protecting [65], also contributed to the positive association between adolescents’ gender egalitarianism and prosocial behavior found in the present study.

In the meantime, adolescents’ prosocial behavior was positively predictive of subsequent gender egalitarianism. This finding is consistent with prior research suggesting the crucial role of behaviors in shaping adolescents’ attitudes and values [5,6]. It provides empirical evidence for the self-perception theory. Specifically, individuals are particularly likely to infer inner attitudes and values by observing their own behaviors when the behaviors, such as prosocial behavior, are highly encouraged by the value system in which they live [46,66,67]. By engaging in frequent prosocial behaviors in daily life, adolescents may become more aware of the similarities between males and females rather than the differences, such as both having times of sadness and vulnerability and needing help from others. In another word, prosocial behavior facilitates the elimination of inter-group bias [11]. This process hence promotes the formation of adolescents’ gender egalitarianism. In summary, the longitudinal interrelationship between adolescents’ gender egalitarianism and prosocial behavior forms a virtuous circle, highlighting the dynamic interplay between the two.

### 4.3. Nonsignificant Moderating Effect of Gender

This study did not find a moderating effect of adolescent gender on the bidirectional associations between gender egalitarianism and prosocial behavior; in other words, such associations applied equally to boys and girls. This gender parity may be due to the homogeneity of the socialization process of boys and girls in China. Given the previous one-child policy, the adolescents in this study were predominantly only children. As the only child or one of the few children in the family, the adolescents’ parents generally provided abundant care and had the same socialization goals for them, regardless of gender [68], such as educational aspiration [69]. Under this circumstance, the boys and girls in the study sample might have experienced similar socialization processes regarding sex roles and prosocial development. Accordingly, despite the levels of gender egalitarianism and prosocial behavior among the adolescents varied across genders, the interplay of the two over time applied equally to the boys and girls.

### 4.4. Limitations and Future Directions

First, this study focused on Chinese adolescents who were socialized in a relatively gender-equal culture, and thus more culturally inclined to treat other people seriously and rightly, regardless of gender. Consequently, the bidirectional links between adolescent gender egalitarianism and prosocial behavior might be culturally specific. It would be preferable to test the generalizability of the current findings in more culturally diverse samples. Second, we focused on the moderating effect of the agent’s gender, while existing research has highlighted the role of the target’s gender in prosocial behavior [70], which allows for more in-depth research. Consequently, it would be advantageous in future research to incorporate the target’s gender when investigating the associations between adolescent gender egalitarianism and prosocial behavior. Third, the present study completed data analysis for three-wave data with the cross-lagged panel model (CLPM), which might not failsafe against backdoor confounders and colliders. If feasible, future studies can benefit from using a dynamic panel model with more waves of data to replicate the findings.

## 5. Conclusions

This research has investigated the bidirectional associations between adolescent gender egalitarianism and prosocial behavior, and the moderating effect of gender in the associations, as well as gender differences and longitudinal changes in both. Drawing on three waves of data over three years, we found that girls reported higher gender egalitarianism than boys; girls reported more prosocial behavior than boys in the sixth grade, but there were no significant gender differences in the seventh and eighth grades. Adolescents’ gender egalitarianism stayed stable from the sixth to the seventh grade then increased from the seventh to the eighth grade, and there was a decrease in prosocial behavior from the sixth to the seventh grade. More importantly, the results showed that adolescents’ gender egalitarianism in the previous year positively predicted prosocial behavior in the next year, and vice versa; such bidirectional associations equally applied to boys and girls, and these longitudinal findings present rank-order changes in individuals rather than within-person changes.

This research makes contributions and has practical implications. At the theoretical level, it supports basic value theory, social dominance theory, and self-perception theory by suggesting the essential role of adolescent gender egalitarianism in prosocial behavior, and vice versa. At the empirical level, it advances our understanding of adolescent gender egalitarianism and prosocial behavior from a developmental perspective. At the practical level, the interplay of values and behaviors as found in this study suggests that optimizing adolescent gender egalitarianism via prosocial behavior interventions, such as organizing students to participate in volunteer activities, and promoting adolescent prosocial behavior via gender-egalitarianism-related interventions, such as engaging students in the discussion surrounding gender issues, may be effective strategies for positive youth development.

## Figures and Tables

**Table 1:** Items of gender egalitarianism and prosocial behavior.

Variable	Item
Gender egalitarianism	“Less important for a woman to work than for a man” (reverse-coded)
	“If both work full-time, man should take equal share of chores”
	“Women should have same training/career chances as men”
	“Should be more women bosses in important jobs”
	“Men and women should have chance to do same kind of work”
Prosocial behavior	“I help my friends, even if it is not easy for me”
	“I really enjoy doing small favors for my friends”
	“I go out of my way to cheer up my friends when they seem sad”
	“I voluntarily help my friends”
	“I always listen to my friends talk about their problems”
	“I enjoy being kind to my friends”
	“I watch out for my friends”

**Table 2:** Measurement invariance across gender and time for the study variables.

Variable	Model	χ^2^	*df*	*p*	CFI	TLI	RMSEA	ΔCFI	ΔRMSEA
Gender invariance for gender egalitarianism	1. Configural invariance	232.78	144	<0.001	0.967	0.952	0.048	/	/
	2. Weak invariance	267.85	156	<0.001	0.958	0.944	0.051	0.009	0.003
	3. Strong invariance	366.14	168	<0.001	0.926	0.908	0.066	0.041	0.180
	4. Partial strong invariance	278.30	162	<0.001	0.957	0.944	0.052	0.01	0.004
Time invariance for gender egalitarianism	1. Configural invariance	157.90	72	<0.001	0.966	0.951	0.047	/	/
	2. Weak invariance	173.09	80	<0.001	0.963	0.952	0.046	0.003	0.001
	3. Strong invariance	208.19	90	<0.001	0.954	0.946	0.049	0.012	0.002
	4. Partial strong invariance	200.51	89	<0.001	0.956	0.948	0.048	0.01	0.001
Gender invariance for prosocial behavior	1. Configural invariance	612.81	360	<0.001	0.966	0.960	0.051	/	/
	2. Weak invariance	636.86	378	<0.001	0.965	0.961	0.050	0.001	0.001
	3. Strong invariance	665.66	396	<0.001	0.964	0.962	0.050	0.002	0.001
Time invariance for prosocial behavior	1. Configural invariance	327.85	180	<0.001	0.980	0.976	0.039	/	/
	2. Weak invariance	340.88	192	<0.001	0.980	0.978	0.038	0	0.001
	3. Strong invariance	400.47	206	<0.001	0.973	0.973	0.042	0.007	0.003

**Table 3:** Descriptive statistics and correlations of the study variables.

Variable	1	2	3	4	5	6
1. Gender egalitarianism at Time 1						
2. Gender egalitarianism at Time 2	0.41 ***					
3. Gender egalitarianism at Time 3	0.41 ***	0.41 ***				
4. Prosocial behavior at Time 1	0.31 ***	0.27 ***	0.20 ***			
5. Prosocial behavior at Time 2	0.18 ***	0.31 ***	0.20 ***	0.43 ***		
6. Prosocial behavior at Time 3	0.24 ***	0.26 ***	0.36 ***	0.39 ***	0.42 ***	
*M*	3.83	3.83	4.01	4.23	4.14	4.20
*S.D.*	0.80	0.84	0.80	0.80	0.75	0.76

*Note*. The analytic sample included 543 participants for Time 1, 449 participants for Time 2, and 417 participants for Time 3. *** *p* < 0.001.

## Data Availability

The data presented in this study are openly available at OSF: https://osf.io/8kepj/?view_only=33ca1a7f2c1f4d4ca5b3bb4509f276e5 (accessed on 16 June 2023).
